# Surround inhibition can instantly be modulated by changing the attentional focus

**DOI:** 10.1038/s41598-017-19077-0

**Published:** 2018-01-18

**Authors:** Yves-Alain Kuhn, Martin Keller, Benedikt Lauber, Wolfgang Taube

**Affiliations:** 1 Movement and Sport Sciences, Department of Medicine, University of Fribourg, Fribourg, Switzerland; 2 Department of Sport Science, University of Freiburg, Freiburg, Germany

## Abstract

To further investigate the mechanism of surround inhibition (SI) and to determine whether adopting different attentional strategies might have an impact on the modulation of SI, the effects of adopting an external (EF) or internal focus of attention (IF) on SI and motor performance were investigated. While performing an index flexion with either an EF or IF, transcranial magnetic stimulation was applied at various time points in 14 healthy subjects. When adopting an EF compared to an IF, the results show an improved motor performance (+14.7% in MVC) and a reduced bEMG in the adjacent APB (−22.3%) during maximal index flexion. This was accompanied by an increased SI in the APB with an EF (+26.4%). Additionally, the decrease in bEMG correlated with the magnitude of SI in APB. The current results demonstrate an efficient way to modulate SI by changing the attentional focus in healthy subjects and might, at least in part, explain the better motor performance being associated with an EF. The present findings help to better understand the positive mechanisms of an EF on SI in the healthy motor system and may also points towards a treatment strategy in pathologies with disturbed SI such as focal hand dystonia.

## Introduction

Surround inhibition (SI) is a physiological mechanism that shapes neuronal activity in both sensory^1^ and motor systems^2,3^. Thus, SI reflects the capacity of excited or active neurons to reduce the activity of the surrounding neurons. In the human motor system, SI can be demonstrated by applying supra-threshold transcranial magnetic stimulation (TMS) over the primary motor cortex (M1) and measuring the amplitude of the resulting motor evoked potential (MEP) in the adjacent muscles^2^. In this specific context, SI within the primary cortex is expressed as the selective reduction in the MEP amplitude in the surrounding muscles before and during a contraction of the target muscle compared to a rest condition. By shaping neural drive during voluntary movements, SI in M1 is supposed to reflect the summation of inhibitory influences on the representation of surrounding muscles^2,4–6^ and is therefore considered to be essential for skilled motor behaviour^3^. For example, it was shown that the force level has an influence on the modulation of SI^7^. More precisely, it was demonstrated that the level of SI increased at low-force levels but was absent at force levels higher than 40% of MVC, strengthening the assumption that SI is primarily involved in the generation of skilled motor tasks. This is further supported by a study of Beck and Hallett^8^, showing that SI is greater and appears earlier when task complexity is increased. Apart from the task, the phase of the movement also influences the level of SI. Surround inhibition is more pronounced during the preparation and initiation phase of a movement but is dramatically reduced or absent during the hold phase of a ramp-and-hold contraction^4^. Furthermore, it is widely accepted that certain pathologies can influence SI. In contrast to healthy individuals, patients suffering from focal hand dystonia (FHD)^4,9^ or Parkinson’s disease^10^ show much lower levels of SI. Disturbances of SI in the cortical motor system of those patients result in a reduced contrast of active target neurons and supposedly inactive neurons that are not directly involved and thereby lead to an overflow of muscle activation in surrounding and usually non-active muscles^4^.

However, little is known about how the activity of the SI-network can be modulated in healthy subjects and in patients. Previous research has demonstrated that SI can be strengthened^11,12^ or weakened^13,14^ by motor training resulting in down- or upregulated MEP amplitudes in the non-trained adjacent muscles. However, in professional musicians, SI was shown to be significantly reduced^14,15^. The reduction in SI was assumed to favor functional coupling in order to facilitate synergistic finger movements^13,14^. However, at the same time, reduced SI has previously been shown to make these experts more prone to pathological states such as dystonia^16^. Thus, for both healthy subjects and patients it seems important to know how to (instantly) modulate SI and how this relates to behavioural outcome measures.

It has been demonstrated that populations with low levels of intracortical inhibition demonstrate impaired motor control in general such as dexterity^17^ or interlimb coordination^18^. In this regard, the relation between intracortical inhibition and motor performance when adopting either an external (EF) or an internal focus of attention (IF) has previously been investigated^19^. Interestingly, when participants were verbally asked to adopt an EF, it was shown that they could prolong sustaining submaximal isometric contractions with their index finger (time to task failure) and that the activity of inhibitory intracortical circuits within M1 (expressed as SICI and subTMS-induced EMG suppression) was instantly enhanced contrasted to when using an IF strategy. Thus, manipulating the focus of attention did not only have a positive influence on motor performance but also had a direct and immediate effect on cortical inhibition within M1. However, this modulatory effect of intracortical inhibition was outlined in the prime-mover and the effects on surrounding muscles were not tested. According to previous research^3,4^, it seems that the local inhibitory GABAergic circuits within M1 play a crucial role in the generation of SI. This hypothesis was supported by showing that FHD patients do not only have a reduction in SI leading to abnormal motor execution, but also display a loss of inhibition on multiple levels of the central nervous system including SICI^4,20,21^.

Based on these previous findings, it was hypothesized that changing the attentional focus may be a powerful tool to influence SI in M1, especially as it was previously suggested that SICI and SI might share common mechanisms^4,22,23^. Thus, adopting an EF might inhibit surrounding muscles what would reflect a focused neural drive and therefore enhanced movement efficiency. Accordingly, the present study aimed to investigate the immediate influence of different attentional strategies (EF vs IF) on (i) motor performance when executing maximal voluntary contractions (MVC) of the first dorsal interosseous muscle (FDI) and (ii) on the amount of SI within M1 influencing the activity of the adjacent muscle APB.

## Methods

### Subjects

Fourteen right-handed subjects (22–35 years; 3 women) agreed to participate in this study. All subjects were free from any known orthopaedic or neurological disorders. They gave their informed written consent before the experiments, which were approved by the local ethics committee (Commission cantonale d'éthique de la recherche sur l'être humain, CER-VD, Switzerland) and were in accordance with the Declaration of Helsinki.

### Recording

**Figure 1:**
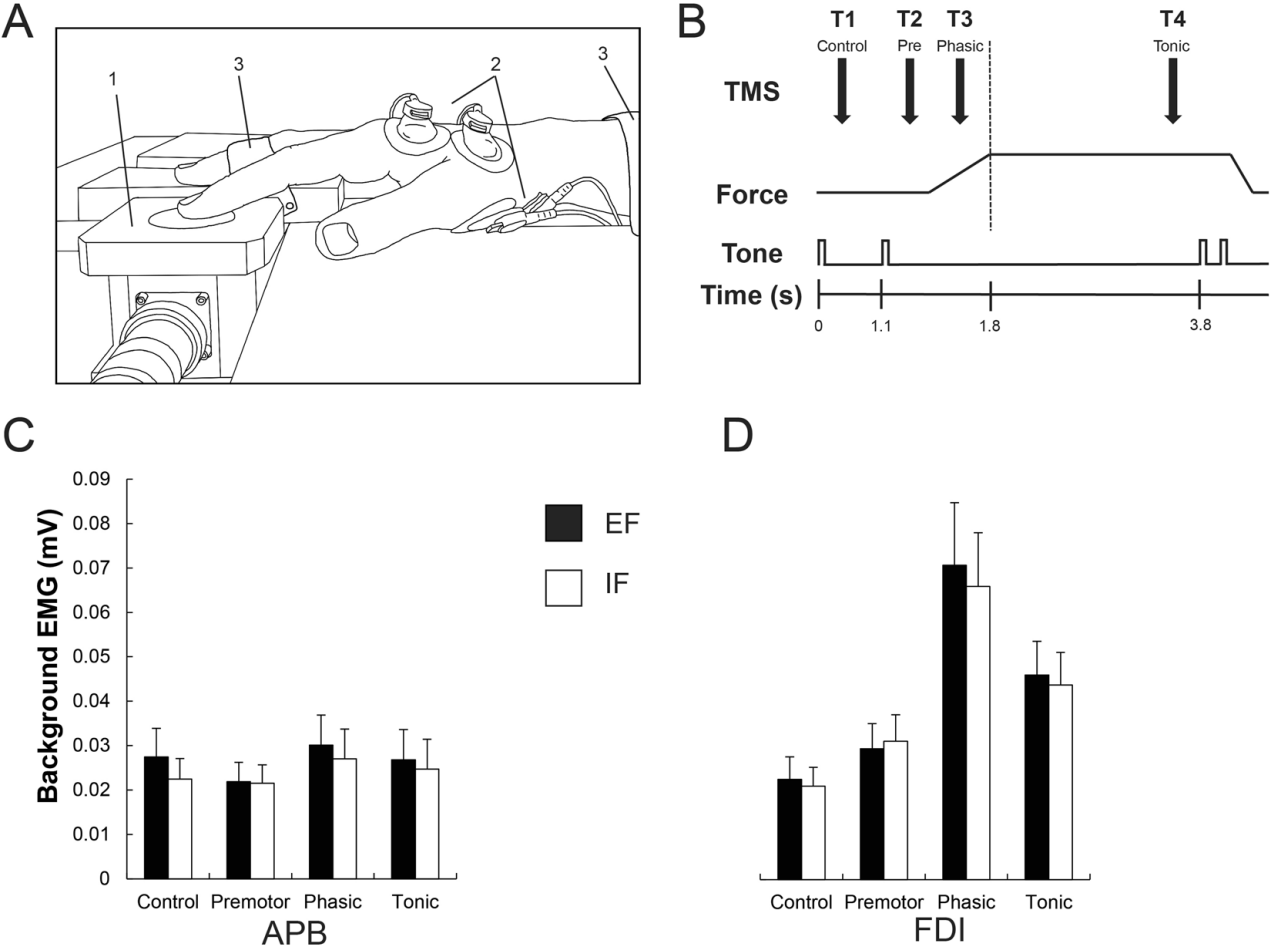
(**A**) Experimental setup. The tip of the right index finger is resting on the force transducer (1). Two pairs of surface electrodes were placed over the (2) APB and FDI. The right forearm, middle, ring and pinky fingers were fixed by straps in order to minimize movements (3). (**B**) Time course of the task. Shown are the transcranial magnetic stimulation (TMS) time points, the force produced and the acoustic tones. Brain stimuli were applied during a resting or control phase (T1; 500 ms after the first tone), during the premotor phase (T2; between 100 and 200 ms after the second tone), the phasic phase (T3; between 300 and 600 ms after the second tone) and the tonic phase (T4; 2700 ms after the second tone). (**C**,**D**) Shown are the background EMG levels, obtained in a 50 ms time window before the brain stimulation, for the surrounding muscle APB (**C**) and the prime mover FDI (**D**) in all four different phases (control, premotor, phasic and tonic). There was a significant main effect of phase in the prime mover FDI, reflecting its activation during the motor task and no significant modulation of background EMG was found in the surrounding muscle APB. Black bars represent the external (EF) and the whites ones the internal focus of attention (IF).

All tests were conducted in one laboratory session. During this session, participants were seated in a comfortable and adjustable chair in an upright position. After skin preparation, bipolar surface electrodes (BlueSensor P, Ambu A/S, Ballerup, Denmark) were placed on the right hand skin over the muscles of interest with 1 cm inter-electrode distance. The reference electrode was also placed on the right hand, on the phalanx of the digitus medius. EMG recordings were obtained from the first dorsal interosseous (FDI) and the abductor pollicis brevis (APB) muscles. EMG recordings were amplified (x 1000), sampled at 4 kHz and bandpass filtered (Butterworth 10–1000 Hz). The individual MEP amplitudes were measured at four different phases of the contraction (see motor task below and Fig. 1B). All data was recorded and stored on a computer for off-line analysis using IMAGO Record software (Pfitec Biomedical Systems, Endingen, Germany).

### Experiment 1

#### Motor task

In experiment 1, subjects were asked to perform maximum isometric contractions by pushing down on a force transducer (MC3A-500; Advanced Mechanical Technologies Inc., Watertown, MA, USA) with the tip of their right index finger in response to an acoustic signal (see Fig. 1A and B). The right forearm, the middle, ring and pinky fingers were fixed by straps in order to minimize all movements. The left arm rested in a comfortable and relaxed position on the table. The force signal was displayed on a computer screen placed 1 m in front them. At the beginning of the session, subjects were asked to perform three MVCs with their right index finger in order to determine the maximal force. Then, after a two minutes break, participants had to perform five MVCs with an IF and, in a second serie, with an EF. The order of the two series was randomized and a five minutes break was given between the two series. Between two consecutive contractions, a rest of 30 seconds was given. During all contractions, EMG and the force signals were recorded.

#### External vs. internal focus of attention

The instruction for the IF condition was ‘*Contract your finger flexor muscles* so that the moving line increases as fast as possible to the maximum after the tone’. For the EF condition, the instruction was ‘*Exert pressure on the force transducer* so that the moving line increases as fast as possible to the maximum after the tone’.

### Experiment 2

In experiment 2, participants were asked to perform an isometric ramp-and-hold contraction by pushing down with their right index finger on the same force transducer as in experiment 1. In response to an acoustic signal, participants were asked to match a moving line, which represented the actual force level, with a horizontal target line. The target line represented 10% of the MVC and was individually adjusted based on the results obtained in experiment 1 (MVC without any specific focus). Ten percent of MVC was chosen based on previous research showing that SI is more pronounced when executing a contraction at low-force levels^7^. The target line and the force signal (running line) were displayed on a computer screen placed 1 m in front of the participants. A first tone indicated the start of each trial and a period of rest that lasted for 1100 ms. Participants were instructed to react to a second tone that was given 1100 ms after the first tone. Thus, they had to increase their force level from zero to 10% of MVC within 700 ms. As participants reacted to the second tone, the onset of force occurred approximately 150–250 ms later. Once the target line was matched, they had to hold the contraction for at least 2000 ms and then returned to the relaxed position (see Fig. 1B). This motor task allowed to differentiate specific phases and to deliver single-pulse TMS during each of these phases (see Fig. 1B): at rest (the control phase, T1; TMS 500 ms after the first acoustic signal), movement preparation (the premotor phase,T2; TMS between 100 and 200 ms after the second tone), the phasic phase (T3; TMS between 300 and 600 ms after the second tone) and the tonic phase (T4; TMS 2700 ms after the second tone). As a first step, participants had to perform practice trials in order to adjust the timing of the stimulation for the premotor- and phasic-phases. The time points of brain stimulation were individually adjusted in order that T2 coincide with the preparatory phase and T3 with the phasic phase. Once determined, the timing of the stimulation was identical for the IF and EF conditions. After these practice trials, participants had to repeat two series of twenty repetitions of the ramp-and-hold contraction with both an IF and an EF, resulting in eighty stimulation (2 foci (EF vs IF) * 4 time points (T1 vs T2 vs T3 vs T4) * 10 trials). Thus, 10 MEPs were recorded and used in the final analysis for each time-point and focus.

#### External vs. internal focus of attention

The instruction for the IF condition was like in experiment 1: ‘*Contract your finger muscles* so that the moving line displayed on the monitor increases from the baseline to the target value after the tone’. The instruction for the EF condition was ‘*Exert pressure on the force transducer* so that the moving line displayed on the monitor increases from the baseline to the target value after the tone’. After five trials, the participants were reminded to ‘concentrate on your finger muscles’ (IF) or ‘exert pressure on the force transducer’ (EF).

#### Brain stimulation

Transcranial magnetic stimuli were applied to the left M1 using a MagVenture Pro stimulator (MagVenture A/S, Farum, Denmark) with a 95 mm focal figure-of-eight coil (MagVenture D-B80). The initial stimulation point was set approximately 5 mm anterior to the vertex and over the midline. In order to ensure that the induced current flow is approximately perpendicular to the central sulcus, the TMS coil was oriented tangential to the scalp with the handle pointing backwards and laterally at 45° angle towards the contralateral forehead^24^. Induced currents were in the reverse mode (posterior to anterior) and the waveform monophasic throughout the whole experiment. The motor hotspot for eliciting MEPs in the APB with minimal intensity was determined by moving the coil anterior and left from the vertex, while MEP size was monitored. Once found, the APB motor hotspot position was recorded and constantly controlled by a neuronavigation system (Polaris Spectra, Northern Digital Inc., Waterloo, Canada and Localite TMS Navigator Version 2.0.5, LOCALITE GmbH, Sankt Augustin, Germany). Then, the resting motor threshold (rMT) was determined to the nearest of 1% of maximal stimulator output. The rMT was defined as the minimal stimulation intensity required to evoke MEPs bigger or equal to 50 μV peak-to-peak amplitude in 3 out of 5 trials. A stimulation intensity of 140% rMT was used when assessing SI. Ten stimuli at each of the four different phases (rest, premotor, phasic, tonic) were delivered in a randomized order in each condition (EF, IF). The order of conditions was randomized between participants.

### Data analyses and statistics

#### EMG and reaction time analysis

In experiment 1, the maximal force was considered as the highest peak in the force signal (low pass filtered at 50 Hz) during the contraction. In addition, the five trials were averaged and compared between both conditions (EF vs IF). Background EMG (bEMG) was obtained in the FDI (prime mover) and APB (adjacent) muscles and compared by assessing the root mean square in a time window of 100 ms following the peak of force (in experiment 1) and 50 ms preceding the brain stimulation (in experiment 2). In addition, the power spectrum density function of the EMG signal during the MVCs was computed by a fast Fourier transformation. The mean (MNF) and median frequencies (MDF) were used as the parameter to compare the power spectrum between both focus of attention conditions (EF vs IF). The MNF was computed as the mathematical mean of the spectrum curve and the MDF as the parameter that divides the total power area into two equal parts. Finally, the reaction time (RT) was compared between both conditions as the time from the second acoustic signal – indicating the participants to press against the force transducer in order to reach the target line representing the 10% of the MVC – to the onset of force.

#### Motor evoked potentials analysis

In experiment 2, the peak-to-peak amplitude of the TMS evoked MEPs at each time points (control, premotor, phasic and tonic) in the APB and FDI muscles were compared between both conditions (EF vs IF). Additionally, the correlation coefficient was computed using a robust regression model to investigate association between the modulations of bEMG in the APB muscle (experiment 1) and SI (experiment 2). A robust regression model was applied as it is recommended when data might be contaminated with influential observations and/or outliers^25^. Differences in APB bEMG and MEPs (related to the control phase in percent) used for the regression analysis were obtained by subtracting the value found in the EF condition from the value found in the IF condition. The bEMG in the APB muscle was taken from the first experiment as participants strongly activated their FDI in order to fulfil the task and consequently, only in this experiment, it was difficult for the subjects to not co-activate with their APB muscle. In contrast, FDI activation in experiment 2 was not challenging enough to elicit any co-activation in the adjacent APB.

#### Statistics

Before the statistical analyses, normal distribution of the data was tested using a Shapiro-Wilk test. In experiment 1, separate paired Student *t*-tests were performed for each output parameter of the behavioural tests (force, bEMG and power spectrum analysis) to compare the two conditions (EF vs. IF). In experiment 2, neurophysiological outcome measures (peak-to-peak MEP amplitudes and bEMG) were compared individually using a two-way repeated measures ANOVA to compare the effect of “focus” (two levels: EF and IF) and the effect of “phase” (four levels: control, premotor, phasic and tonic). If sphericity was violated (Maulchy’s test), degrees of freedom were corrected by Greenhouse-Geisser estimates of sphericity. Effect sizes are presented as general eta-squared values^26^ and the Benjamini-Hochberg procedure was used to correct for multiple comparisons in case of significant *F* values^27^. Unless indicated otherwise, data are reported as mean ± standard deviation. R version 3.2.4^28^ was used for all statistical analyses and the level of significance was set at *P* ≤ 0.05.

### Data availability

The datasets generated during the present study are available from the corresponding author on reasonable request.

## Results

### Experiment 1

**Figure 2:**
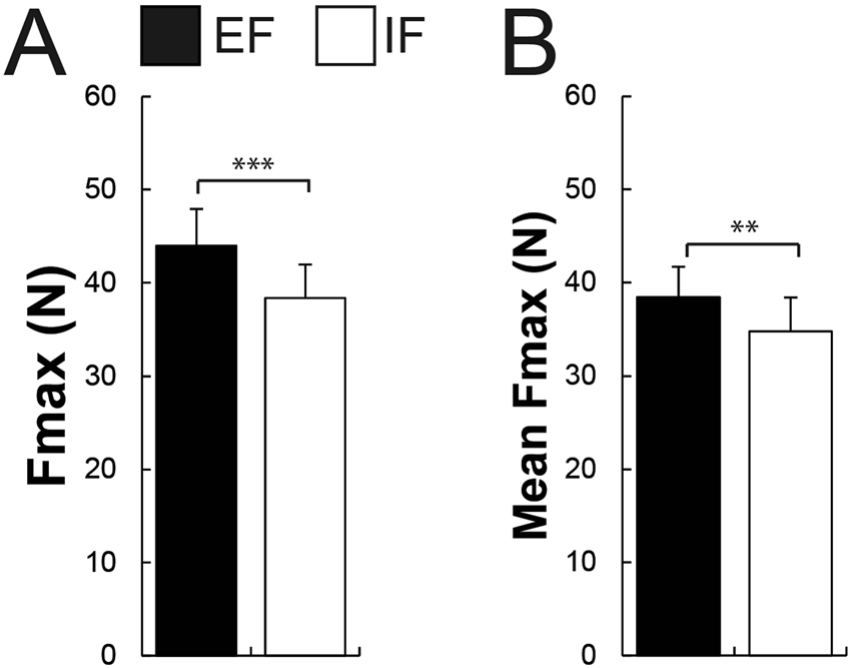
Shown are mean values and SEM (*n* = 14) of (**A**) the force during maximal voluntary contraction (MVC, the best trial) and (**B**) the force during the 5 MVC trials averaged under both attentional focus conditions (EF vs IF). Participants could generate more force and more force when the 5 MVC trials were averaged with an EF compared to an IF. Black bars represent the external (EF) and the whites ones the internal focus of attention (IF). **P ≤ 0.01, ***P ≤ 0.001.

**Figure 3:**
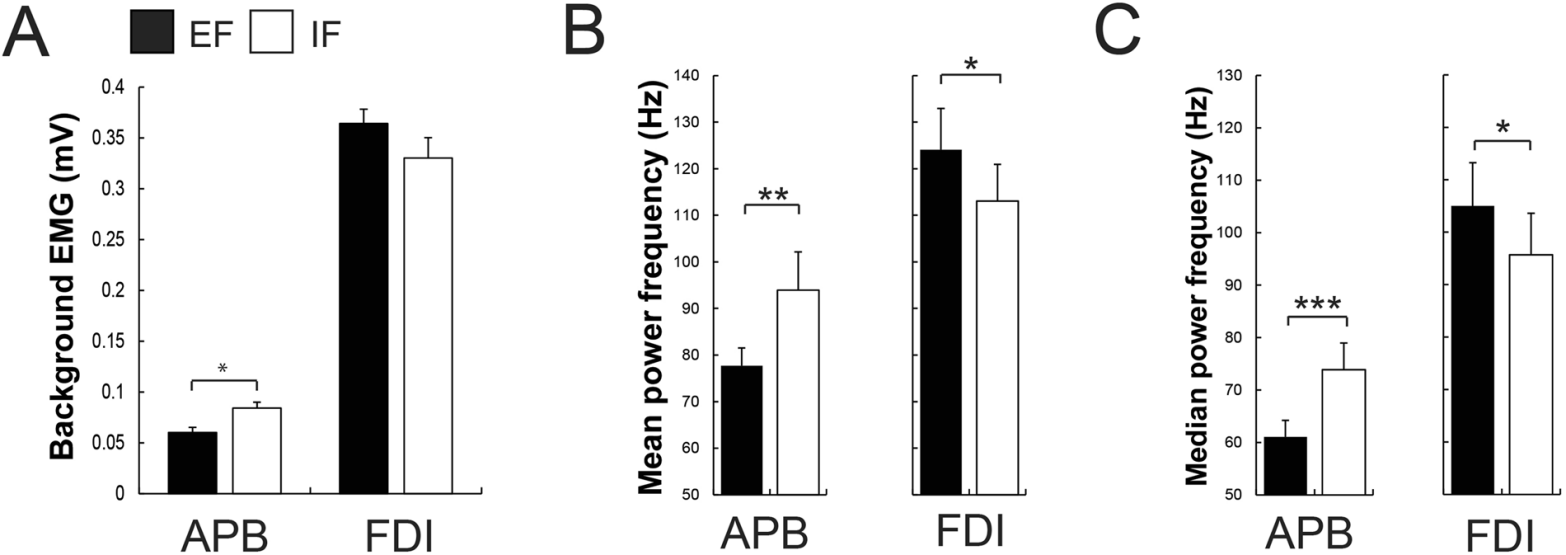
In all bar graphs, shown are mean values and SEM (*n* = 14). (**A**) Participants showed less background EMG during the MVC in the adjacent APB muscle when focusing externally (EF) compared to focusing internally (IF) while no significant difference was found in the prime mover FDI muscle. (**B**) Shown are the mean power frequency (MNF) and (**C**) median power frequency (MDF) of adjacent APB and prime mover FDI muscles during the MVC under both focus of attention conditions. Participants showed a smaller MNF and MDF in the APB muscle and a greater MNF and MDF in the prime mover FDI when adopting an EF contrasted to an IF. Black bars represent the external (EF) and the whites ones the internal focus of attention (IF). *P ≤ 0.05, **P ≤ 0.01, ***P ≤ 0.001.

Participants could generate more force (the best of the 5 trials) when adopting an EF (43.98 ± 14.76 N) compared to an IF (38.34 ± 13.53 N; *t*_13_ = 5.46, *P* < 0.001, Fig. 2). In addition, they displayed more force on average (the 5 trials averaged) with an EF (38.43 ± 12.23 N) contrasted to an IF (34.75 ± 13.65 N; *t*_13_ = 3.77, *P* = 0.002, Fig. 2). Additionally, subjects showed less bEMG activity in the adjacent muscle APB (*t*_13_ = −2.48, *P* = 0.02, Fig. 3A) when executing the maximal force task with an EF (0.059 ± 0.05 mV) compared to an IF (0.076 ± 0.07 mV). Additionally, they showed smaller MNF and MDF in the adjacent APB muscle when adopting an EF contrasted to an IF (MNF: *t*_13_ = −2.99, *P* = 0.01, EF = 77.67 ± 13.28 Hz, IF = 93.95 ± 28.39 Hz; MDF: *t*_13_ = −3.99, *P* = 0.001, EF = 61.05 ± 10.92 Hz, IF = 73.87 ± 17.67 Hz, Fig. 3B and C). Regarding the bEMG of the prime mover FDI between both conditions, no significant difference was found (*P* = 0.14, Fig. 3A). However, participants displayed significant greater MNF and MDF when focusing externally compared to internally (MNF: *t*_13_ = 2.53, *P* = 0.02, EF = 124.07 ± 30.66 Hz, IF = 113.04 ± 27.30 Hz; MDF: *t*_13_ = 2.41, *P* = 0.03, EF = 105.01 ± 28.57 Hz, IF = 95.66 ± 27.54 Hz, Fig. 3B and C).

### Experiment 2

#### Brain stimulation paradigm

The same subjects as in experiment 1 took part in the second experiment. However, two participants showed a very inconsistent motor performance during this second experiment and were therefore excluded from the final data analysis.

**Figure 4:**
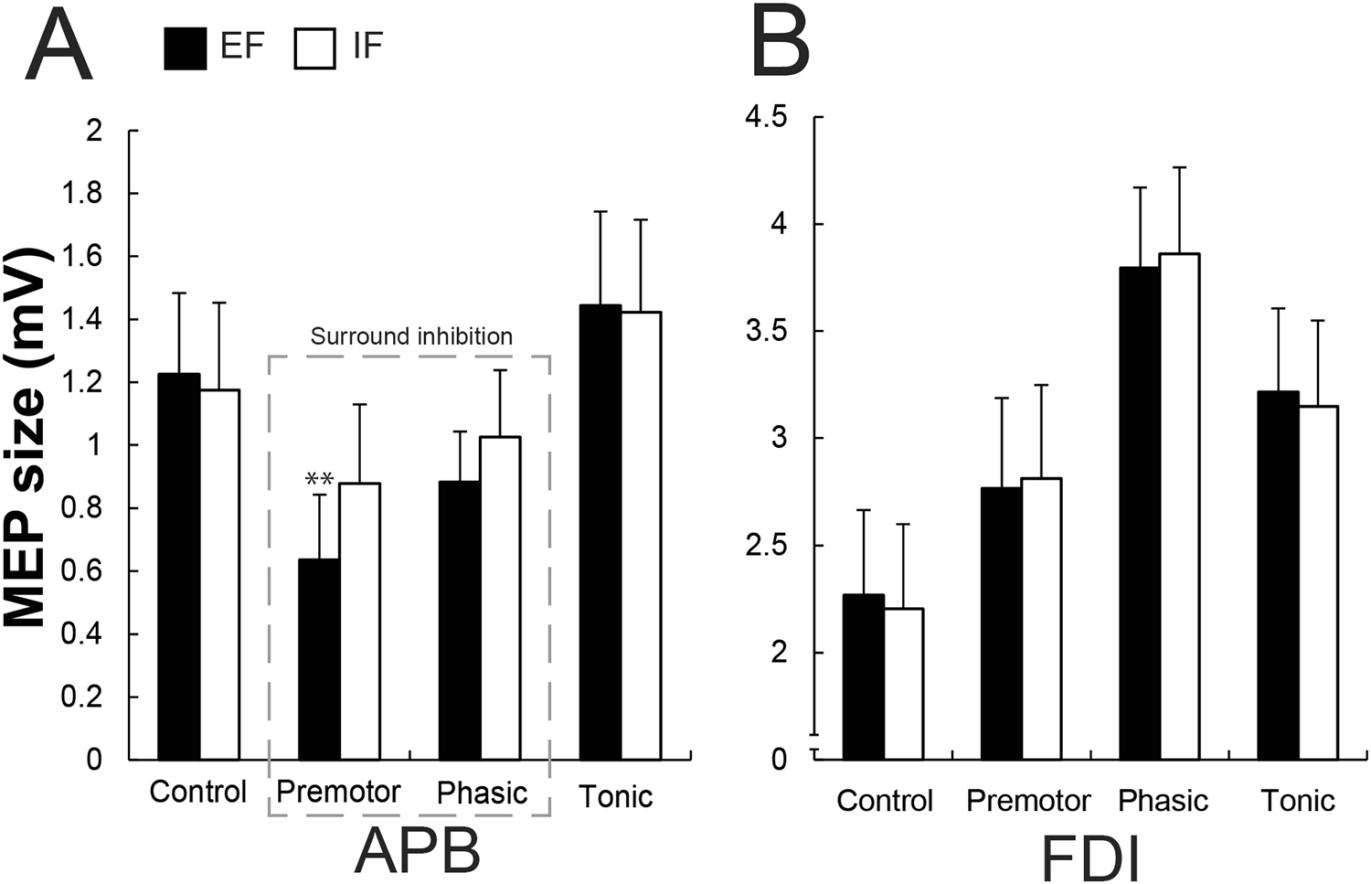
(**A**,**B**) Shown are mean values and SEM (*n* = 12) of the MEP peak-to-peak amplitude in the two surrounding muscle APB (**A**) and the prime mover FDI (**B**) at four different brain stimulation time points (Control, Premotor, Phasic and Tonic) under the two attentional focus conditions (EF and IF). In the surrounding muscle APB, there was a significant interaction effect showing that when adopting a EF contrasted to an IF, surround inhibition was enhanced during the premotor phase. There was a significant main effect of phase in the prime mover FDI (**B**), reflecting its activation during the motor task. Black bars represent the external (EF) and the whites ones the internal focus of attention (IF). **P ≤ 0.01.

When analysing MEP peak-to-peak amplitudes in the APB muscle, there was no main effect of focus (*F*_1,11_ = 3.75, *P* = 0.07, *η*^2^ = 0.002) but a significant main effect of phase (*F*_1.92,21.12_ = 9.30, *P* = 0.001, *η*^2^ = 0.086). Additionally, a significant interaction effect of focus x phase was found (*F*_3,33_ = 7.18, *P* < 0.001, *η*^2^ = 0.005). Pairwise *Post hoc* comparisons for the main effect of phase revealed that the premotor phase (*P* < 0.001) and phasic phase (*P* = 0.04) were smaller than the control phase and that all phases differed from the tonic phase (all *P* < 0.001, see Table 1 for more details). Regarding the interaction effect of focus x phase, the pattern of modulation of MEP size in the APB muscle for the different phases showed a significant reduction in MEP size (reflecting the SI) before the initiation of the index finger flexion (premotor) compared to the control phase (*P* = 0.001 in the EF and *P* = 0.04 in the IF condition). Moreover, a clear enhancement in MEP size during the tonic phase when compared to all other phases (all *P* < 0.04, see Table 1 for details) was shown. Most importantly, MEP sizes during the premotor phase were significantly smaller when adopting an EF contrasted to an IF (*P* = 0.004), indicating a differential SI of the MEP in the APB regarding the type of attentional focus (Fig. 4A). No significant inhibition was found during the phasic phase compared to the control condition for both attentional foci (*P* = 0.10 in the EF and *P* = 0.38 in the IF condition, respectively).

**Table 1:** Single-pulse TMS in surrounding muscle APB for all four phases of the movement and the two conditions

	**EF**				**IF**			
	**Control**	**Premotor**	**Phasic**	**Tonic**	**Control**	**Premotor**	**Phasic**	**Tonic**
MEP size (mV)	1.22 ± 0.89	0.63 ± 0.71a,b,c	0.88 ± 0.55a,b,e	1.44 ± 1.03a,c,d	1.17 ± 0.96	0.87 ± 0.86a,b,d,f,g	1.02 ± 0.73a,b,g	1.42 ± 1.02a,f
MEP (% of control MEP)	100	45.30 ± 17.92	79.69 ± 44.82	121.76 ± 29.73	100	71.69 ± 27.04	95.79 ± 47.31	130.76 ± 39.03
bEMG (mV)	0.024 ± 0.02	0.021 ± 0.01	0.028 ± 0.02	0.021 ± 0.02	0.019 ± 0.01	0.020 ± 0.01	0.017 ± 0.02	0.017 ± 0.02

Shown are mean values and SD (*n* = 12) for single-pulse MEP size, MEP size related to control MEP expressed in percent and the background EMG (bEMG) for both conditions (EF and IF) in the surrounding muscle APB. Main effect of phase: significant difference (*P* < 0.05) for this phase in both conditions compared with the control phase (a) and compared with the tonic phase (b). Interaction effect focus x phase: significant difference (*P* < 0.05) for this phase compared to control (c), premotor (d), tonic phase (e) in the EF condition and compared to control (f) and tonic phase (g) in the IF condition. EF = external focus, IF = internal focus of attention.

In the prime-mover FDI muscle, the MEPs were recorded with the coil over the APB hotspot. There was no main effect of focus (*F*_1,11_ = 0.01, *P* = 0.91, *η*^2^ < 0.001) but a significant main effect of phase (*F*_2.03,22.37_ = 12.60, *P* < 0.001, *η*^2^ = 0.15, Fig. 4B). Pairwise *post hoc* comparisons revealed that all phases differed from the control phase (premotor, *P* = 0.04; phasic, *P* < 0.001; tonic, *P* = 0.001, Table 2 for more details). Additionally, MEPs were significantly enhanced compared to the premotor (*P* < 0.001) and tonic phases (*P* = 0.001) during the phasic phase. Furthermore, MEPs in the tonic phase were significantly bigger contrasted to the premotor phase (*P* = 0.002). There was no significant interaction effect of focus x phase (*F*_3,33_ = 2.81, *P* = 0.054, *η*^2^ < 0.001, Fig. 4B). Due to the fact that the *P* value was close to significance threshold, we performed additional separate paired Student’s *t*-tests (uncorrected), to test whether differences between both conditions (EF vs IF) occurred for any phase. Actually, no significant difference was found between the conditions (EF vs IF) in the control phases (*t*_11_ = −1.19, *P* = 0.25), premotor phases (*t*_11_ = 0.92, *P* = 0.37), phasic phases (*t*_11_ = 0.85, *P* = 0.41), and tonic phases (*t*_11_ = −1.59, *P* = 0.13).

**Table 2:** Single-pulse TMS in the prime mover (FDI) for all four phases of the movement and the two conditions.

	**EF**				**IF**			
	**Control**	**Premotor**	**Phasic**	**Tonic**	**Control**	**Premotor**	**Phasic**	**Tonic**
MEP size (mV)	2.26 ± 1.37	2.76 ± 1.45a	3.79 ± 1.29a,b	3.21 ± 1.35a,b,c	2.20 ± 1.36	2.81 ± 1.50a	3.86 ± 1.39a,b	3.14 ± 1.38a,b,c
MEP (% of control)	100	133.13 ± 78.48	206.42 ± 117.50	165.45 ± 90.64	100	138.13 ± 69.54	214.84 ± 111.80	164.98 ± 79.30
bEMG (mV)	0.022 ± 0.01	0.029 ± 0.01	0.07 ± 0.04	0.046 ± 0.02	0.021 ± 0.01	0.031 ± 0.02	0.066 ± 0.04	0.043 ± 0.02

Shown are mean values and SD (*n* = 12) for single-pulse MEP size, MEP size related to control MEP expressed in percent and the background EMG (bEMG) for both conditions (EF and IF) in the prime mover FDI muscle. Main effect of phase: significant difference (*P* < 0.05) for this phase in both conditions compared with control phase (a), compared with the premotor phase (b), and compared with the phasic phase (c). No interaction effect of focus x condition were found in FDI. EF = external focus, IF = internal focus of attention.

#### Background EMG and reaction time

Background EMG recordings in the APB muscle revealed no significant main effect of focus (*F*_1,11_ = 2.28, *P* = 0.15, *η*^2^ = 0.004), main effect of phase (*F*_1.26,14.19_ = 0.86, *P* = 0.39, *η*^2^ = 0.015) and no interaction effect of focus x phase (*F*_1.56,17.16_ = 0.65, *P* = 0.58, *η*^2^ = 0.001, Table 1 and Fig. 1C).

In the prime mover FDI muscle, there was no significant main effect of focus (*F*_1,11_ = 1.78, *P* = 0.20, *η*^2^ < 0.001). However, there was a significant main effect of phase (*F*_1.47,16.17_ = 14.36, *P* < 0.001, *η*^2^ = 0.28). *Post hoc* comparisons revealed that background EMG in all phases was bigger than the control phase (premotor, *P* = 0.01; phasic, *P* < 0.001; tonic, *P* < 0.001), showing that FDI was active during the movement (see Fig. 1D). Additionally, background EMG was significantly enhanced during the phasic (*P* < 0.001) and tonic (*P* = 0.003) phases compared with the premotor phase. Background EMG was significantly enhanced in the phasic phase compared to the tonic phase (*P* < 0.001, Table 2 for details). No significant interaction effect of focus x phase (*F*_3,33_ = 1.36, *P* = 0.27, *η*^2^ = 0.001) was found.

Regarding the RT (the onset in the force signal after the second tone), no significant difference was found between both conditions (*t*_11_ = −0.95, *P* = 0.35; EF = 198.11 ± 37.77 ms and IF = 203.32 ± 35.08 ms).

### Correlation

**Figure 5:**
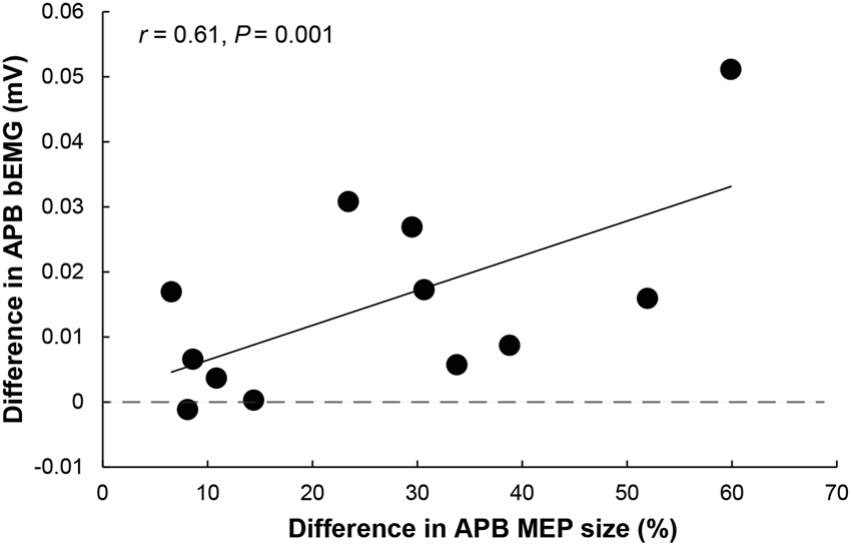
Shown are the attentional foci-related differences (IF–EF) of APB MEPs size during the premotor phase (related to control as percentage changes) on the abscissa and the differences (IF–EF) in APB bEMG during maximal contraction of the index finger on the ordinate. The robust regression model clearly demonstrates that greater decrease in bEMG found in the adjacent muscle APB during maximal contraction of the prime mover FDI (experiment 1) is accompanied by greater surround inhibition during the premotor phase in the adjacent APB (experiment 2, *r* = 0.61, *P* = 0.001).

A potential connection between the functional data (experiment 1) and the neurophysiological data (experiment 2) was tested applying a robust regression model. The difference in the bEMG of the adjacent muscle APB between EF and IF in experiment 1 was related to differences in SI between EF and IF in experiment 2. The results showed a significant coefficient of correlation between the difference in bEMG in the APB muscle during the maximal contraction and the amount of SI in the APB muscle during the premotor phase (*r* = 0.61, *P* = 0.001, Fig. 5).

## Discussion

The current results from experiment 2 demonstrate an efficient way to instantly modulate SI by changing the focus of attention. Furthermore, as demonstrated by experiment 1, adopting an EF resulted in an improved motor performance and a reduced muscular activity in the adjacent muscle APB. As the decreased bEMG found in the APB muscle during maximal index finger contraction showed a significant correlation with the enhanced SI found during the premotor phase, the improved motor performance that is usually observed with an EF may at least partly be explained by an enhanced activation of neural circuits that shape motor SI.

### Attentional foci and motor performance

In the present study, experiment 1 was designed (a) to confirm that motor performance can be enhanced when adopting an EF and (b) to assess (unwanted) muscular activity in the adjacent muscle APB.

In line with previous research (see^29^ for a review) and as expected, motor performance was improved when adopting an EF contrasted to an IF. More specifically, asking the participants to concentrate on the force transducer they were pushing against (EF), rather than on their index finger (IF), led to an enhanced maximal force of the index finger (+14.7%). At the same time and in line with previous research, subjects revealed less bEMG activity (−22.3%), MNF and MDF in the adjacent APB muscle with an EF^30^. Furthermore, despite no significant difference in bEMG between conditions in the prime-mover FDI, participants showed significantly larger power spectral density in the FDI muscle, expressed as enhanced MNF and MDF, when focusing externally.

As the force a muscle can produce depends not only on the number of motor units activated (recruitment following the size principle), but also on the rates at which motor neurones discharge action potentials (rate coding)^31^, this enhanced frequency found in the FDI muscle under an EF might be explained in at least two ways: first, additional motor units (MUs) might have been recruited that fire with higher frequencies and/or the already active MUs have increased their firing frequency. The first mechanism would suggest that maximal force production with an IF is not able to fully recruit all MUs and that the upward shift in the MNF and MDF with an EF in the prime mover FDI is indicative of an additional recruitment of MUs that, according to the size principle^32^, consist of larger MUs with faster conduction velocities^33^. The second mechanism does not necessarily involve recruitment of additional MUs but may be due to the fact that an increased incidence of doublet discharges occurred at the very beginning of the MVC. The presence of doublet discharges at the onset of muscle contraction known as the catch-like property of skeletal muscle^34^ is thought to improve force production^35,36^ and might, therefore, help to generate more force with an EF.

In addition, our results confirm previous findings showing reduced muscular activity with an EF^30,37–42^. Based on this observation, it was previously argued that an EF increases movement efficiency by reducing unnecessary muscular contributions^30^. Therefore, our results suggest that adopting an EF contrasted to an IF modified the pattern of muscle activation (less activation of the adjacent muscle APB coupled with an upward shift in the power spectral density in the FDI) during the motor task and led to an increased MVC. However, the underlying mechanisms for this focus-specific EMG reduction in the adjacent muscle remains generally poorly understood and, therefore, experiment 2 was designed.

### Attentional foci and motor surround inhibition

In experiment 2, we investigated the influence of changing the focus of attention on the amount of SI in the APB muscle. Similar to previous studies^4,7,8^, a reduction in the amplitudes of APB MEPs in the premotor and phasic phases during the index finger flexion occurred, indicating the presence of SI in this adjacent muscle. In the present study, the amount of SI during the movement initiation phase could be instantly altered by changing the focus of attention: in both the premotor (+26.4%; *p* = 0.004) and the phasic phase (+8.1%; non-significant change), adopting an EF resulted in a greater SI than an IF. Furthermore, the advantage of the present study is that we could compare one and the same subject performing the identical task with an EF or IF (using a repeated measures design) in contrast to the first study investigating the underlying mechanism of adopting different attentional foci by means of fMRI^43^.

### Surround inhibition and hand motor control

So far, the relationship between motor behaviour and SI is a key but unanswered part in understanding the role of SI in hand motor control and performance. As SI is more prominent with low-force levels and disappears at intensities higher than 40% of MVC, it seems that SI is predominantly involved in the generation of skilled and fine motor tasks^8^. Additionally, previous research showed that FHD patients demonstrate unusual co-contractions, which are thought to be associated with a reduced SI during movement initiation of the index finger^4^. Thus, it is argued that an enhanced SI improves the contrast on the motor cortical level and thereby facilitates dynamic manual tasks^3^. In addition, motor training was shown to not only improve motor performance but also to strengthen SI^11,12^.

The observations of the current study may suggest that the increased efficiency usually associated with an EF on a behavioral level^29,44,45^ may in fact be due to differential neural activation of motor areas such as M1: as shown in experiment 2, the increased SI seems to focus the motor command to the prime mover, avoiding – as shown in experiment 1 – unnecessary contractions of muscles that are not directly involved. The finding of a correlation between the decrease in bEMG and increase in SI in the APB further strengthens this notion. The current results in the adjacent APB also complement our previous study concentrating on the prime mover FDI. In this previous study, increased levels of SICI and sub-TMS induced EMG suppression with an EF were shown for the agonist FDI^19^. Thus, adopting an EF seems to influence motor cortical inhibitory control for both, the prime mover as well as the surrounding muscles that are not directly involved in the motor task.

It was formerly suggested that SICI might contribute to the inhibitory network shaping SI^4,22,23^. Indeed, previous research revealed a reduction of short-interval intracortical inhibition (SICI) in dystonic patients^2,9,46^ accompanied by an impaired SI in the premotor and phasic phase of the movement^4^. Thus, it seems that these inhibitory processes are both closely interrelated and influence the quality of motor execution in general^4^. Nonetheless, at this point, the link between SI and SICI remains unclear and further research is needed.

### Limitations of the current study

Although the present study demonstrates an instant modulation of motor cortical SI when changing the focus of attention, it cannot clarify whether SI is created in M1 or if it is derived from input coming from other brain areas such as the basal ganglia^3^ and/or cerebellum^47^. For example, in patients with impaired SI, the basal ganglia circuits have been assumed to display imbalanced activity between direct – responsible for sculpting the desired movement –, and indirect pathways – responsible for inhibiting unwanted movements^3^. In addition, FHD patients show an hyper-activation of cerebellum in fMRI during voluntary movement, which might be related to the disrupted SI^47^. Therefore, the finding of the present study cannot render more precisely these underlying mechanisms but shed more light on the interrelation between behavioural changes and modulation in SI.

### Conclusion and functional considerations

The current study provides new evidence that SI can be instantly modulated according to attentional foci in healthy individuals. Importantly, the increase in SI is associated with better motor performance. Therefore, the instant modulation of SI induced by verbal instructions leaves open the possibility of influencing SI in a therapeutic setting, when abnormal SI is thought to impair motor function in neurological conditions such as FHD^3^.
